# On the Mechanism of Magnesium Storage in Micro- and Nano-Particulate Tin Battery Electrodes

**DOI:** 10.3390/nano8070501

**Published:** 2018-07-06

**Authors:** Francisco Nacimiento, Marta Cabello, Carlos Pérez-Vicente, Ricardo Alcántara, Pedro Lavela, Gregorio F. Ortiz, José L. Tirado

**Affiliations:** Departamento de Química Inorgánica e Ingeniería Química, Instituto Universitario de Investigación en Química Fina y Nanoquímica IUIQFN, Universidad de Córdoba, Campus de Rabanales, Edificio Marie Curie, E-14071 Córdoba, Spain; q92nacof@uco.es (F.N.); z22cabbm@uco.es (M.C.); iq3pevic@uco.es (C.P.-V.); iq2alror@uco.es (R.A.); iq1lacap@uco.es (P.L.); iq1ticoj@uco.es (J.L.T.)

**Keywords:** electrode nanomaterials, magnesium-tin intermetallics, magnesium-ion batteries, Sn-119 Mössbauer spectroscopy

## Abstract

This study reports on the electrochemical alloying-dealloying properties of Mg_2_Sn intermetallic compounds. ^119^Sn Mössbauer spectra of β-Sn powder, thermally alloyed cubic-Mg_2_Sn, and an intermediate MgSn nominal composition are used as references. The discharge of a Mg/micro-Sn half-cell led to significant changes in the spectra line shape, which is explained by a multiphase mechanism involving the coexistence of c-Mg_2_Sn, distorted Mg_2−δ_Sn, and Mg-doped β-Sn. Capacities and capacity retention were improved by using nanoparticulate tin electrodes. This material reduces significantly the diffusion lengths for magnesium and contains surface SnO and SnO_2_, which are partially electroactive. The half-cell potentials were suitable to be combined versus the MgMn_2_O_4_ cathodes. Energy density and cycling properties of the resulting full Mg-ion cells are also scrutinized.

## 1. Introduction

Recent concerns regarding the future availability of lithium (Li) [1,2,3,4], together with safety issues [5] affecting Li-ion batteries have prompted expanding research activity on alternatives to lithium. Due to its high abundance, easy extraction and moderate cost, magnesium (Mg) is one of the multivalent elements that show promising possibilities [6,7,8,9,10,11,12,13]. Despite its lower potential compared with lithium, Mg electrodes provide higher volumetric capacity and shows a better reconstruction of its surface on cycling, being less prone to form dendrites on electroplating. Mg^2+^ ions have a similar radius to Li^+^ ones and avoid pronounced volume changes on cycling. However, there is a major difficulty in obtaining reliable Mg-anode batteries, related with the stability of electrolyte solutions [12,13]. Thus, electrolytes commonly stable versus Mg are mostly unstable versus common cathodes found so far and vice versa. For this reason, the research for alternative anodes and hence the concept of Mg-ion batteries is also valid for this alkali-earth element. Recently, the chemical and electrochemical Mg deinsertion from MgMn_2_O_4_, which leads to Mg_1−*x*_Mn_2_O_4_ or λ-MnO_2_, has been reported using both aqueous and non-aqueous electrolytes [14,15,16]. While examining the possible use of several materials as anodes to be combined vs. MgMn_2_O_4_ as a positive electrode, the most successful results were found for tin (Sn), a material previously reported to have reversible electrochemical alloying reactions with Mg [17]. To study these alloying reactions, we apply ^119^Sn Mossbauer spectroscopy (MS), a uniquely selective technique, to study changes in the oxidation state and chemical environment of tin. When tin oxides and intermetallic compounds are used as active electrode material in lithium batteries, ^119^Sn MS provides valuable information [18,19,20,21,22]. Particularly, Aldon et al. found ^119^Sn MS useful to study lithium insertion in c-Mg_2_Sn [21].

A ^119^Sn Mössbauer study is reported here for Sn powdered electrodes in Mg test cells, to unveil the details of the complex mechanism of the electrochemical reaction, which involves a tin-rich intermetallic phase with an electric field gradient environment of tin atoms and cubic Mg_2_Sn. Also, due to the increasing interest in nanomaterials for battery applications [23,24], the optimization of the electrode was carried out by using a nano-dispersed Sn-SnO*_x_* composite powder that provides a unique surface electroactive coating of tin oxides, allowing for better cycling stabilities. Finally, the nano-tin electrodes are combined with a low temperature MgMn_2_O_4_ material recently reported by our research group to obtain a novel suitable Mg-ion battery.

## 2. Materials and Methods 

Commercial magnesium strip (purity ≥ 99%, Sigma-Aldrich Química S.L., Madrid, Spain) and micro- and nano-Sn (Aldrich) powders were used as received. Thermally prepared Mg-Sn intermetallic samples included stoichiometric crystalline Mg_2_Sn and a sample with MgSn nominal stoichiometry. Both thermal samples were obtained from mechanical mixtures of Mg and micro-Sn, which were heated at 700 °C for 1 h and then cooled to room temperature at 4 °C min^−1^ in a N_2_ atmosphere. The low-temperature spinel (LT)-MgMn_2_O_4_ was prepared as described elsewhere [16], following the Pechini method. The dry powdered precursor was first heated at 200 °C, ground in an agate mortar and then heated at 400 °C for ten hours. 

The electrochemical experiments were performed in a multichannel VMP instrument (Bio-Logic, Barcelona, Spain). Swagelok-type cells were mounted in an M-Braun glove-box filled with Ar. Tin powders and magnesium strips were used as received. The working electrodes were a mixture of active material:carbon black:polyvinylidene difluoride (PVDF) binder in a 80:10:10 ratio supported on Ti substrate. The carbon black additive was supplied by Société des Accumulateurs Fixes et de Traction (S.A.F.T., Bordeaux, France). The electrode mass load was 3.0–5.0 mg cm^−2^. Several non-aqueous electrolyte solutions were tested, including 0.5 M PhMgCl in tetrahydrofuran (THF) or 0.5 M EtMgCl in THF for Mg/Sn half cells, and 0.5 M Mg(ClO_4_)_2_ in acetonitrile (AN) for full cells. Mg-ion full cells were tested in Swagelok™-type three-electrode cells to monitor separately cathode and anode voltages versus a reference electrode consisting of a metallic Mg disk.

X-ray diffraction (XRD) measurements were carried out in a Bruker D8-Advance instrument (Bruker Española S.A., Madrid, Spain) with CuK_α1_ radiation. Ex-situ XRD patterns of discharged electrodes were recorded by dismantling the electrochemical cells in the dry box under Ar atmosphere and, after recovering the electrodes and separating them from the Ti collector, covering them with a Kapton bag to avoid contact with air.

The ^119^Sn Mössbauer spectra (MS) were recorded in a WissEl instrument (WissEl-Wissenschaftliche Elektronik GmbH, Starnberg, Germany) at room temperature. The spectra were recorded with adequate acquisition time to permit a deconvolution, typically ten days. The ^119^Sn isomer shifts are referenced to BaSnO_3_. A pure β-Sn foil was used for the calibration. For the fitting of the experimental spectra, the WINSO1.0 program (F. Landry, P. Schaaf, WinISO: Windows Mössbauer Fitting Program, unpublished), Lorentzian line-shape absorption peaks, and a least-squares method were employed. When the fitting process reached the convergence, the quality of the fitting was controlled by the classical χ^2^-test. The Mössbauer spectra of discharged electrodes were recorded ex-situ by putting the active material under Ar atmosphere in polybags (Aldrich), which were hermetically closed by heat-sealing with a commercial heat sealer at 150 °C.

Field-Emission Scanning Electron Microscope (FESEM) images were obtained in JEOL FESEM (Izasa Scientific, Madrid, Spain) 1400 provided with Energy-dispersive X-ray spectroscopy (EDX).

## 3. Results

**Figure 1:**
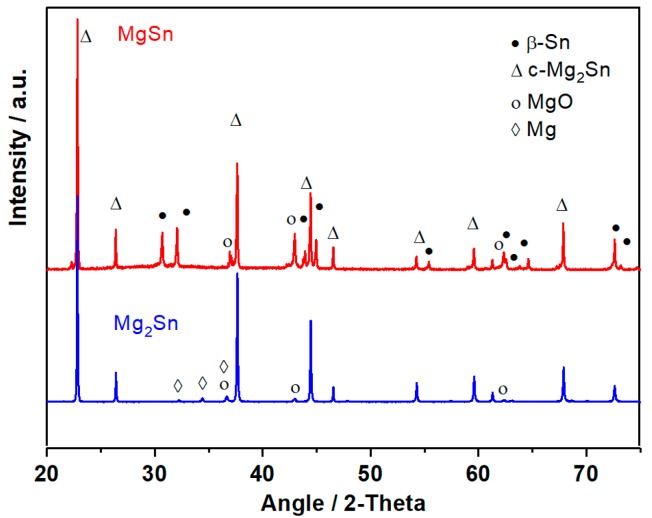
Powder X-ray diffraction (XRD) patterns of the thermally prepared samples with Mg_2_Sn and MgSn nominal compositions.

Figure 1 shows the X-ray diffraction patterns of thermally prepared, crystalline cubic c-Mg_2_Sn, a fluorite-type structure, and the thermally prepared MgSn material, which contains both crystalline β-Sn and c-Mg_2_Sn with visible traces of MgO impurities and unreacted Mg. 

**Figure 2:**
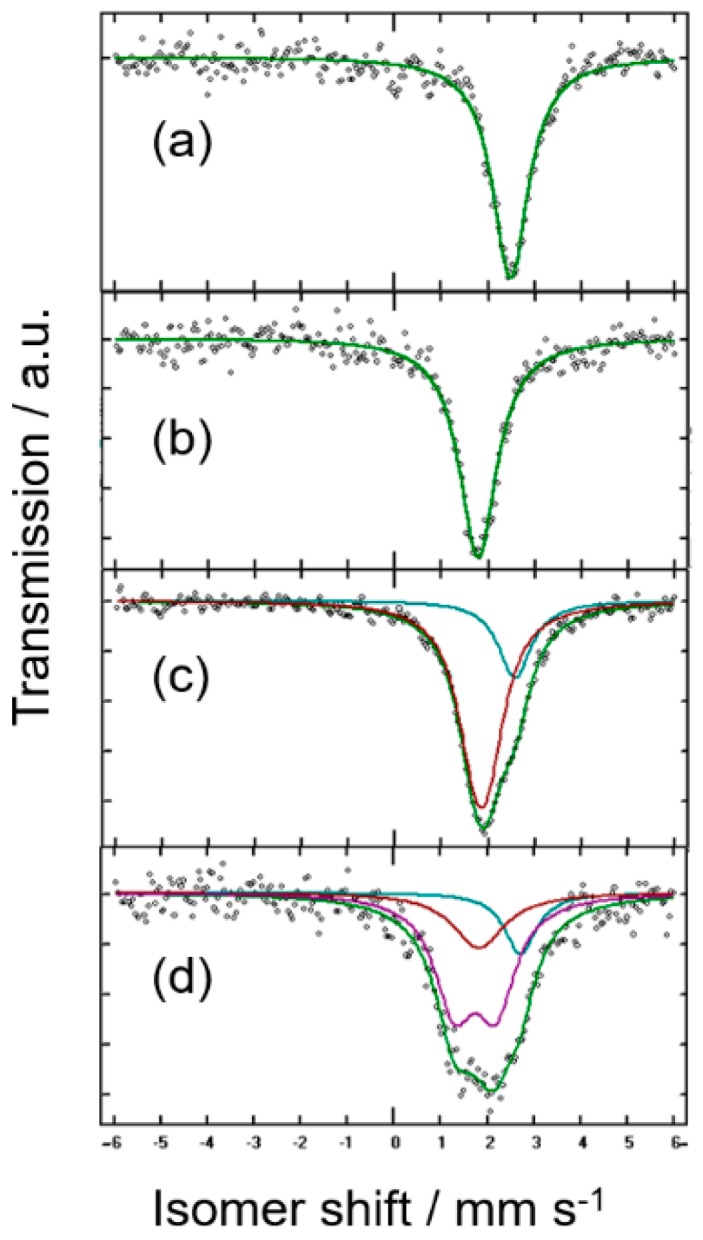
^119^Sn Mössbauer spectra of (**a**) tin microparticles, and thermally prepared samples with (**b**) Mg_2_Sn and (**c**) MgSn nominal compositions. (**d**) micro-Sn electrode after discharge in Mg half-cell to a Mg_0.4_Sn nominal composition.

Figure 2a shows the ^119^Sn MS data for the commercial tin microparticles. The observed isomer shift (IS) value of 2.561_9_ mm s^−1^ (Table 1) and its negligible quadrupolar splitting are consistent with a high-purity and well crystallized β-Sn phase. 

Figure 2b shows the spectrum of the crystalline Mg_2_Sn alloy. The IS value of 1.847_9_ mm s^−1^ is ascribable to cubic c-Mg_2_Sn [21,25]. In agreement with the well-known fluorite-type structure of c-Mg_2_Sn, tin atoms are eight-fold coordinated by magnesium and twelve-fold coordinated by tin second neighbors, which is in contrast with the use of less and more directional covalent bonds in β-Sn and agrees with the significantly lower isomer shift relative to β-Sn (Figure 2a). Again, the highly symmetric coordination of tin atoms in the structure impedes a quadrupole splitting of the signal (Table 1). The spectrum of thermal MgSn shows two deconvoluted contributions close to c-Mg_2_Sn and β-Sn-related phases (Figure 2c). However, this spectrum reveals unexpected results. Thus, the singlets ascribable to crystalline β-Sn and c-Mg_2_Sn were not sufficient to fit the spectrum. A significant quadrupole splitting (0.30_4_ mm s^−1^) of the low IS signal was also present (Table 1). This visually unresolved doublet is indicative of the presence of tin nuclei in a low symmetry environment of Sn atoms. It is probably due to incomplete coordination by Mg atoms, in a metastable, non-stoichiometric and distorted d-Mg_δ_Sn phase, which is not discerned from the crystalline products in the XRD patterns. This result is consistent with the report by Sirkin et al. [26] on quenched ternary Sn-Mg-M alloys, and latter corroborated by theoretical calculations by Fries and Lukas [27].

**Figure 3:**
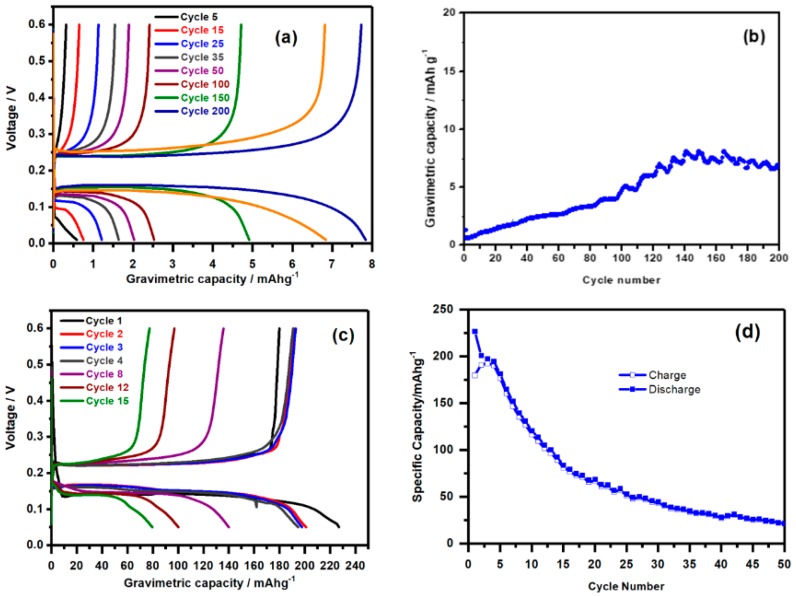
(**a**) Galvanostatic charge-discharge plots and (**b**) cycling performance at at 10 mA/g (C/20) current density of microparticulate β-Sn in Mg half-cell, using 0.5 M PhMgCl in THF as electrolyte. (**c**) Galvanostatic charge-discharge plots and (**d**) cycling performance at 10 mA/g (C/20) of nanoparticulate-Sn in in Mg half-cell using 0.5 M EtMgCl in THF as electrolyte.

Figure 3a,b shows the cycling properties of the Mg/0.5 M PhMgCl (THF)/micro-Sn cell. Extremely low capacity values were obtained in the 0.01–0.6 V potential window, although the capacity increases upon cycling (<10 mA h g^−1^), probably indicating the progressive conditioning of the metal electrode surface. According to the Gibbs phase rule, the presence of well-defined plateaus in both discharge and charge should be consistent with a biphasic mechanism of the alloying-dealloying reaction:

β-Sn + 2Mg^2+^ + 4e^−^ ⇌ c-Mg_2_Sn (Theoretical capacity 903 mA h g(Sn)^−1^) (1)

The reaction mechanism of the micro-Sn electrodes was explored by using ^119^Sn MS. Figure 2d shows the results for a discharged electrode prepared by applying multiple galvanostatic pulses followed by relaxation periods to a Mg/micro-Sn half-cell until a stable nominal Mg_0.4_Sn stoichiometry was achieved. The spectrum shows significant changes in line shape as referred to the pristine tin microparticles (see Figure 2a) that agree with tin electroactivity. The IS parameters shown in Table 1 evidence the simultaneous presence of a Mg-deficient, distorted Mg_2−δ_Sn phase, a β-Sn-related phase with possible Mg doping and c-Mg_2_Sn, which partially agrees with the biphasic mechanism suggested above. The low proportion of c-Mg_2_Sn is indicative of impediments to the full conversion of the tin microparticles, probably due to an incomplete diffusion of magnesium through the larger tin particles. Moreover, the high broadening and significant quadrupole splitting of the d-Mg_δ_Sn signal may point to a structural deterioration and/or partial Mg alloying in a metastable phase yielding many different local environments of the tin atoms. It is well known that electrochemical reactions may lead to metastable products, thus being one of the most useful soft-chemistry synthetic routes for the solid state [18,19,20,21,22].

**Figure 4:**
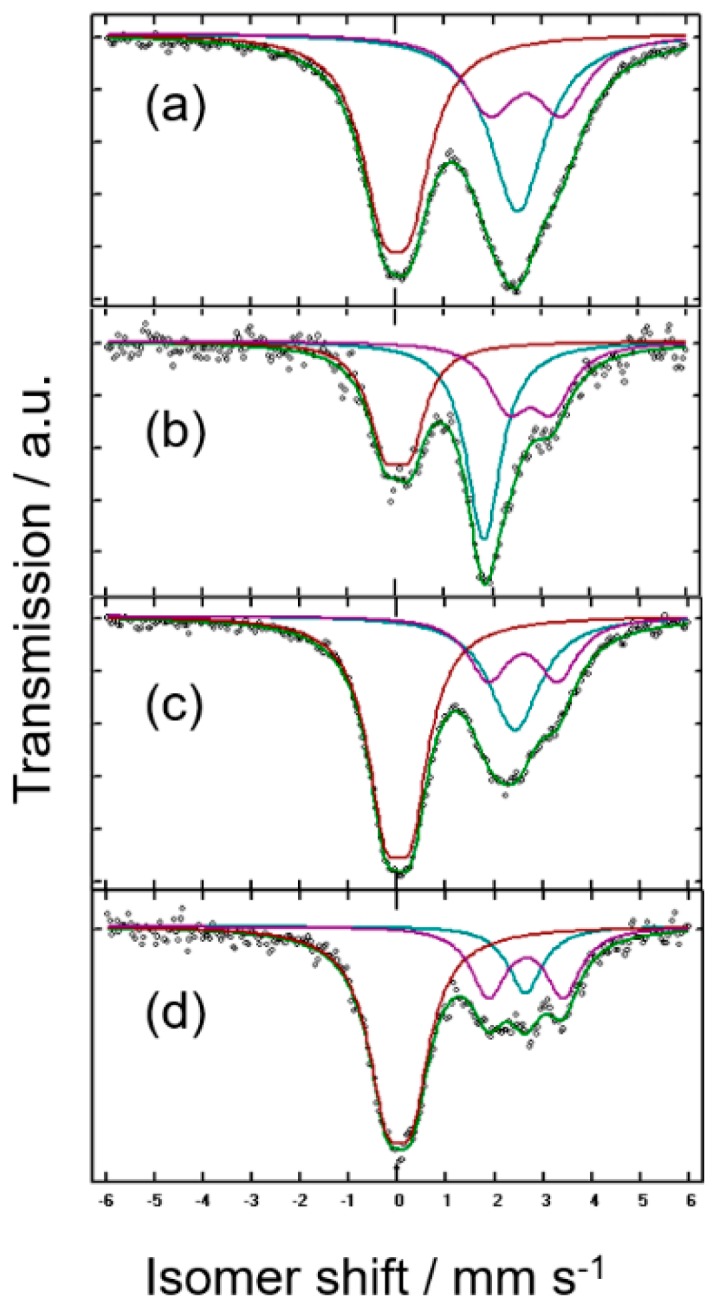
^119^Sn Mössbauer spectra of (**a**) Commercial nano-Sn, and (**b**) discharged nano-Sn electrodes in Mg half-cell to a Mg_1.3_Sn nominal composition. (**c**) Discharged-charged nano-Sn electrodes in Mg half-cell to a Mg_0.4_Sn nominal composition. (**d**) Discharged-fully charged nano-Sn electrodes in Mg half-cell.

**Figure 5:**
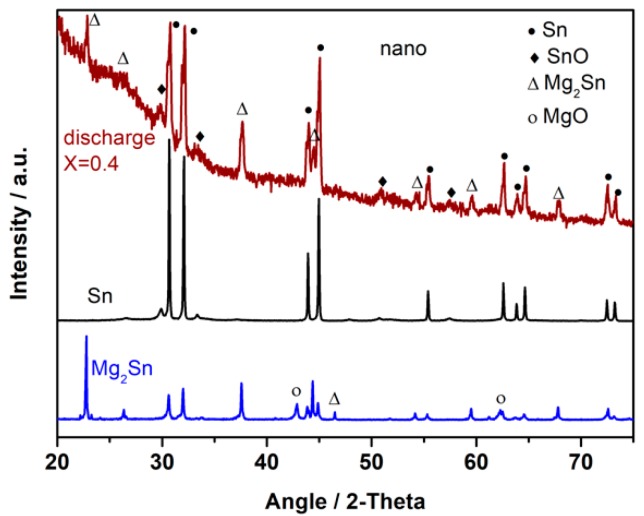
XRD pattern of commercial nano-Sn, and discharged nano-Sn electrode in Mg half-cell at Mg*_x_*Sn nominal compositions with *x* = 0.4 and *x* = 2.0.

**Figure 6:**
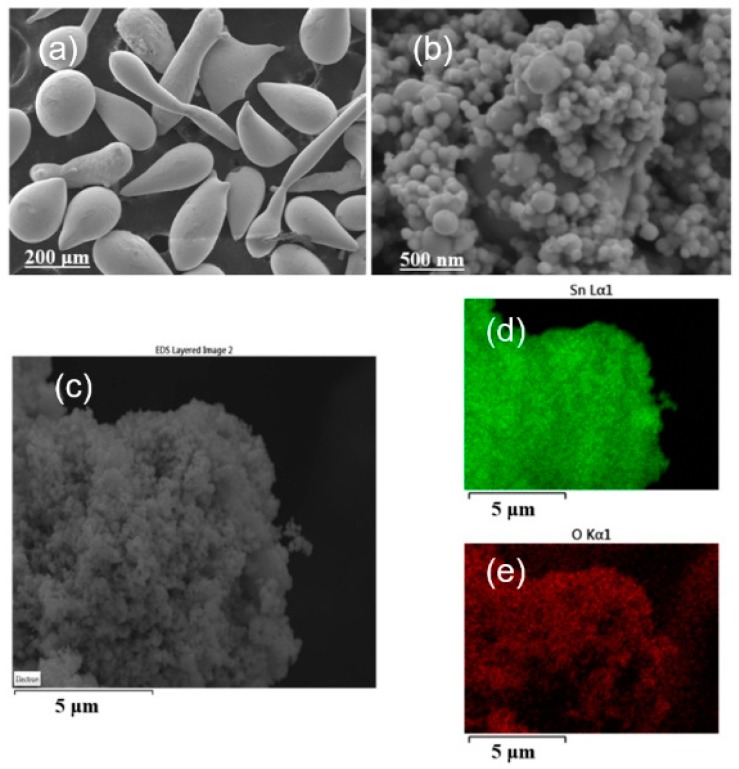
FESEM (Field-Emission Scanning Electron Microscope) images of (**a**) micro-Sn, and (**b**,**c**) nano-Sn. EDX composition mapping of nano-Sn: (**d**) tin and (**e**) oxygen.

To improve the electrochemical performance, a nanodispersed commercial sample (nano-Sn) was also assessed. ^119^Sn MS data were also recorded. This spectrum was deconvoluted in several components attributed to β-Sn, a quadrupole split signal of SnO (IS = 2.65 mm s^−1^), and SnO_2_ with a cassiterite structure with IS ca. 0.0 mm s^−1^ (Figure 4a and Table 2) [28]. The high intensity of the signals attributed to tin oxides, as compared with that of metallic tin is a consequence of the significantly lower *f* value for the latter. The necessary corrections lead to an atomic percentage of 76% β-Sn. The XRD pattern shows reflections of β-Sn and some additional low intensity lines that could be ascribable to SnO (Figure 5) to support this conclusion. The presence of the oxides may involve the surface oxidation of the tin nanoparticles. This process is particularly visible in nanoparticulate materials due to their high surface-to-volume ratio. However, its presence could provide passivation of the electrode material that could prevent undesirable surface reactions with the electrolyte during the cycling or contribute to the total capacity if the oxides are electroactive. Figure 6 shows the FESEM images of micro and nano-Sn. In contrast to the ca. 100 μm particles of crystalline tin, nano-Sn shows particles around 100 nm. Also, the EDX spectra showed an average Sn/O atomic ratio of 1.16, and the composition mapping showed a uniform distribution of oxygen in the surface of the particles. With this in mind, we decided to use the nano-Sn sample without further chemical treatments for the electrochemical experiments. 

Figure 3c,d shows the galvanostatic cycling experiments of Mg/nano-Sn half-cells. The initial capacity (225 mA h g^−1^) is higher and cell polarization is lower than that of micro-Sn cell, preserving well-defined plateaus. However, capacity fades for prolonged cycling, as that of micro-Sn, probably because of the poor response of the Mg metal electrode in the used electrolytes. However, nanodispersion is expected to be a suitable strategy to improve the initial capacity of full Mg-ion batteries, such as the proof of concept discussed below. The average discharge and charge voltages are 0.1 V and 0.25 V, respectively. The limited cell polarization and low charge potential suggest that a Sn/0.5 M PhMgCl (THF)/MgMn_2_O_4_ full cell (ca. 2.0 V) would provide a suitable energy density.

Figure 4b shows ^119^Sn MS data for nano-Sn electrodes after discharge to a nominal Mg_1.3_Sn composition. The fitting parameters in Table 2 reveal the expected formation of c-Mg_2_Sn; however, the tin oxides initially present in the samples are still present, thus offering a sufficient coating of the tin nanoparticles to be preserved during the alloying-dealloying process and stabilize the electrode structure upon cycling. The XRD pattern for *x* = 0.4 in Figure 5 is also in agreement with the MS data. The good reversibility of the process is exemplified by the ^119^Sn MS data for nano-Sn electrodes after recharge to a nominal Mg_0.4_Sn composition (Figure 4c,d and Table 2). Although the oxide lines were always present, it can be highlighted that the initial SnO_2_/SnO ratio in nano-Sn decreases during the discharge and increases again during charge. The SnO_2_/SnO pair can be then considered electroactive, as shown in other systems [28]. Not only does this prevent undesirable surface reactions but it also contributes to the overall capacity.

A better perspective of the possible use of these materials as anodes in Mg-ion batteries can be given by trying their electrochemical behavior in full cells. For this purpose, the cycling properties of the nano-Sn/MgMn_2_O_4_ full cells were tested with different electrolyte solutions. The expected overall reaction can be written as:

2 MgMn_2_O_4_ + *x* nano-Sn ⇌ 2 Mg_1−*x*_Mn_2_O_4_ + *x* Mg_2_Sn (2)

**Figure 7:**
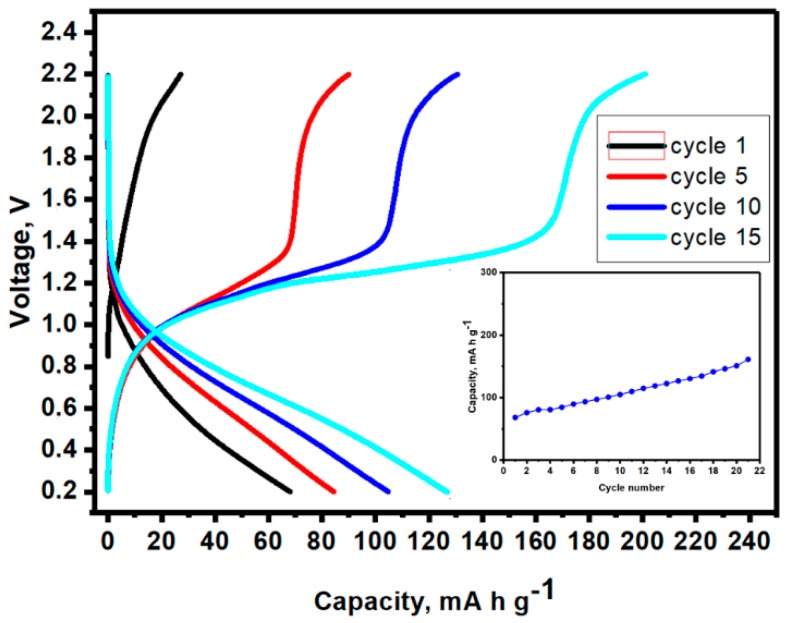
Galvanostatic charge-discharge plot at 20 mA g^−1^ of a nano-Sn/MgMn_2_O_4_ full cell, using 0.5 M Mg(ClO_4_)_2_ in AN electrolyte and m_+_/m_−_ = 4:1. Inset: Capacity vs. cycle number.

Due to the incompatibility of the organometallic electrolytes versus MgMn_2_O_4_ [16], and perchlorate electrolytes in acetonitrile versus Mg metal, we will avoid the use of Mg metal in full cells. Then, the changes in the voltage of nano-Sn/MgMn_2_O_4_ full cell were monitored by using two electrode cells and Mg(ClO_4_)_2_ in AN as the electrolyte. Several mass ratios m_+_/m_-_ were examined. Figure 7 shows the best response observed that corresponds to a spinel mass excess (m_+_/m_−_ = 4.0) that could provide enough magnesium extraction from the cathode during charge to complete Equation (2) in the anode, even if *x* = 1.0. The capacities were thus calculated by using the anode mass. The full Mg-ion cell showed the typical S-shaped voltage profile with an average discharge potential close to 0.8 V and moderate polarization and an increasing discharge capacity up to ca. 150 mA h g^−1^ (Figure 7). This trend is related with the conditioning behavior of the anode that was discussed in the light of Figure 3. An energy density of up to ca. 120 W h kg^−1^ can be estimated considering the mass of the anode.

## 4. Conclusions

The alternative intermetallic anode Mg_2_Sn/Sn for Mg-ion batteries is evaluated in Mg half cells with the valuable help of ^119^Sn Mössbauer spectroscopy. This technique suggests the electrochemical alloying-dealloying properties by a complex mechanism involving amorphous intermetallic phases with an electric field gradient in the environment of tin atoms. The small but significant quadrupolar splitting revealed by the fitting of the spectra of the signal ascribable to the tin-rich phase is indicative of an electric field gradient environment of tin atoms. This is probably due to the presence of lower symmetry environments than in c-Mg_2_Sn, because of an incomplete magnesiation. Thus, the coordination polyhedra of tin in c-Mg_2_Sn (CaF_2_-related structure) are SnMg_8_ cubes. For a no-stoichiometric intermediate, SnMg_8−*x*_ polyhedral are less regular and cause the splitting of the nuclear spin levels of the quadrupolar ^119^Sn nuclei. Nanoparticulate tin was also examined. Tin nanoparticles are surrounded by a SnO/SnO_2_ film due to surface oxidation and are partially reduced during cycling. Their lower diffusion lengths for magnesium improve the initial capacity as compared to tin microparticles, although capacity fading is observed, probably because of the magnesium metal electrodes. Thus, the use of full Mg-ion cells has been tested as a proof of concept. The spinel-related solid, MgMn_2_O_4_, which is known to deintercalate magnesium by chemical and electrochemical means in both aqueous and non-aqueous electrolytes, was found to be compatible with Mg_2_Sn anodes. Cycling properties of the full Mg-ion cells provided voltages around 0.8 V. Capacity values and their retention during cycling were good for the Mg(ClO_4_)_2_-AN electrolyte. In this case, a reversible discharge capacity of ca. 25 mA h g^−1^ and maximum energy density of ca. 120 W h kg^−1^ were observed.

## Figures and Tables

**Table 1:** Isomer shift (IS), quadrupolar splitting (QS), line width (LW), % contribution and attribution of the signals appearing in the ^119^Sn Mössbauer spectra of commercial micro- and nano-Sn, mechanochemically produced Mg_2_Sn. and discharged/recharged electrodes. * Recoilless fractions, *f* = 0.05 (Sn). Reproduced with permission of [28,29]. Copyright Elsevier, 2000, 1966. *f* = 0.3 (c-Mg_2_Sn) Reproduced with permission of [29]. Copyright Elsevier, 1966. *f* = 0.15 (Mg_2−δ_Sn; ca. half of the reported value [29], due to the possible tin excess). These values were used to convert spectral contributions (%) into semiquantitative composition (%_corr_).

Sample (Nominal)	IS/mms^−1^	QS/mms^−1^	LW/mms^−1^	%	%_corr_ *	Attribution
(a) micro-Sn	2.561_9_	-	0.95_3_	100	100	β-Sn
(b) thermal-Mg_2_Sn	1.847_9_	-	0.99_3_	100	100	c-Mg_2_Sn
(c) thermal-MgSn	2.64_3_	-	0.87_7_	24	49	β-Sn
	1.91_3_	0.30_4_	0.96_4_	76	51	Mg_2-δ_Sn
(d) micro-Mg_0.4_Sn	2.79_8_	-	1.83_1_	15	38	β-Sn
discharged	1.8_2_	-	1.4_5_	21	9	c-Mg_2_Sn
	1.79_5_	0.908_2_	1.051_2_	64	53	Mg_2−δ_Sn

**Table 2:** IS, QS, LW, % contribution and attribution of the signals appearing in the ^119^Sn Mössbauer spectra of commercial micro- and nano-Sn, mechanochemically produced Mg_2_Sn and discharged/recharged electrodes. * Recoilless fractions, *f* = 0.05 (Sn and d-Mg_δ_Sn), 0.35 (SnO), 0.60 (SnO_2_), and 0.3 (c-Mg_2_Sn) [28,29]. Copyright Elsevier, 2000, 1966. These values were used to convert spectral contributions (%) into semiquantitative composition (%_corr_).

Sample (Nominal)	IS/mms^−1^	QS/mms^−1^	LW/mms^−1^	%	%_corr_ *	Attribution
(a) nano-Sn	2.579_8_	-	1.41_9_	33	76	β-Sn
	2.75_3_	1.52_5_	1.12_2_	25	2	SnO
	0.026_7_	1.58_1_	1.16_5_	42	13	SnO_2_
(b) nano-Mg_1.3_Sn	1.87_1_	-	0.88_4_	40	50	c-Mg_2_Sn
discharged	2.85_6_	0.90_6_	1.0_1_	27	29	SnO
	0.05_2_	0.53_3_	0.83_6_	33	21	SnO_2_
(c) thermal-MgSn	2.67_2_	-	1.04_3_	14	60	d-Mg_δ_Sn
discharged + charged	1.90_3_	-	1.04_4_	9	7	c-Mg_2_Sn
	2.81_2_	1.22_3_	1.04_1_	23	14	SnO
	0.05_1_	0.55_1_	0.94_2_	54	19	SnO_2_
(d) micro-Mg_0.4_Sn	2.70_8_	-	0.87_4_	15	60	d-Mg_δ_Sn
discharged + fully	2.71_2_	1.57_3_	0.87_7_	26	17	SnO
charged	0.05_1_	0.54_2_	0.96_3_	61	23	SnO_2_
